# Dasatinib-Induced Rhabdomyolysis in a 33-Year-Old Patient with Chronic Myeloid Leukemia

**DOI:** 10.1155/2018/2849869

**Published:** 2018-05-06

**Authors:** Andrew Stevenson Joel Chandranesan, Samip Master, Olivia Antosz, Breanne PeytonThomas, Nebu Koshy

**Affiliations:** ^1^Department of Internal Medicine, Louisiana State University Health Sciences Center, Shreveport, LA, USA; ^2^Department of Pharmacy, University Hospital, Shreveport, LA, USA

## Abstract

Rhabdomyolysis is a life-threatening syndrome due to breakdown of the skeletal muscle. It can be caused by massive trauma and crush injuries or occur as a side effect of medications. Here, we describe a case of a 33-year-old male with human immunodeficiency virus (HIV) and newly diagnosed chronic myeloid leukemia (CML) with severe life-threatening rhabdomyolysis due to a rare offending agent.

## 1. Background

Rhabdomyolysis is a life-threatening syndrome characterized by breakdown of the skeletal muscle. This leads to release of intracellular contents into circulation. Etiology of rhabdomyolysis is diverse, including drugs, toxins, trauma, excessive muscular activity, extreme temperatures, muscle ischemia, infections, and electrolyte abnormalities. Patients' clinical presentation can range from asymptomatic to severe multiorgan dysfunction with renal failure [1]. Tea-colored urine and muscle pain are the common symptoms [2]. Serum creatine kinase (CK) is the most sensitive lab test used for the diagnosis of rhabdomyolysis [3]. One rare cause of rhabdomyolysis is treatment with tyrosine kinase inhibitors (TKIs), which has been illustrated in a few case reports. There has been only one case report of rhabdomyolysis associated with dasatinib in literature [3]. Here, we describe a case of a 33-year-old HIV male with a recent diagnosis of CML with life-threatening rhabdomyolysis due to dasatinib.

## 2. Case Presentation

**Figure 1:**
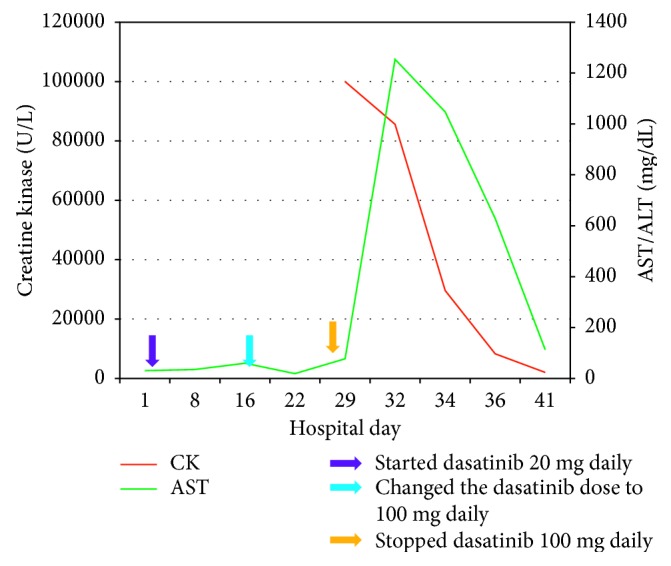
Trend in patient serum creatine kinase (U/L) and serum aspartate aminotransferase (AST) from hospital day 1 to hospital day 41.

A 33-year-old African American male patient was admitted to our hospital as a transfer from an outside hospital for further evaluation of the white blood cell (WBC) count of more than 100 K/mm [3]. He started experiencing fever, chills, poor appetite, and 10-pound weight loss over a month before admission. He visited an outside hospital with these symptoms. Basic laboratory investigations were done which revealed the WBC count of more than 100 K/mm [3]. His past medical history is significant for human immunodeficiency virus infection for the past 10 years. He has been noncompliant with his medications for HIV for more than 6 months. He had an appendectomy in 2003. He denied smoking cigarettes, drinking alcohol, and use of illicit drugs. He worked as a bus driver. He denied any exposure to pet animals or birds. He was afebrile on admission with blood pressure in the normal range. On examination, he had enlarged bilateral cervical, axillary, and inguinal lymph nodes. They were rubbery to touch and not tender on palpation. Absolute CD4 cell count was 847 cells/mm [3] (reference range: 401–1532), and HIV viral load in serum was 101,591 copies/ml (reference range: not detected). Labs on admission showed that white blood cell count was 110.76 K/mm^3^ (reference range: 3.6–11.2 K/mm^3^) with 11% bands, 2% basophils, 29% blasts, 1% eosinophils, 3% lymphocytes, 7% metamyelocytes, 7% myelocytes, 9% promyelocytes, 8% monocytes, and 23% segmented neutrophils. Serum creatinine was 1.4 mg/dL (reference range: 0.7–1.3 mg/dL), alanine aminotransferase (ALT) was 14 U/L (reference range: 12–78 U/L), aspartate aminotransferase (AST) was 31 U/L (reference range: 15–37 U/L), and total bilirubin was 0.5 mg/dL (reference range: 0.2–1.0 mg/dL). He was started on intravenous fluids and allopurinol for mild acute kidney injury from tumor lysis. He was also started on a regimen of emtricitabine-tenofovir 200 mg–300 mg once daily with dolutegravir 50 mg daily for HIV. Bone marrow biopsy and aspiration were performed from the right iliac crest. Complete bone marrow evaluation with bone marrow aspiration, biopsy, and molecular and cytogenetic analysis was done, and it revealed chronic myeloid leukemia in blast crisis (35% bone marrow blasts). The cytogenetic analysis showed t(9:22) and inv(3). BCR-ABL FISH was positive. The RT-PCR was positive for coexpression of p210 and p190 fusion transcripts. He was started on intensive induction chemotherapy on hospital day 4 with cytarabine 100 mg/m [2], continuous intravenous infusion (CIVI) for 7 days with idarubicin 12 mg/m [2], and intravenous infusion (IV) for 3 days (also known as 7 + 3) along with dasatinib. At our institution, we use voriconazole for fungal prophylaxis during intensive induction chemotherapy. Due to drug interactions between dasatinib and voriconazole, the dose of dasatinib was reduced from 100 mg PO daily to 20 mg PO daily [4]. On hospital day 16, it was noted that his total bilirubin was elevated at 1.8 mg/dL (reference range: 0.2–1.0 mg/dL), AST was elevated at 60 (reference range: 15–37 U/L), and ALT was at 41 (reference range: 12–78 U/L). Since voriconazole is metabolized through the liver, it was stopped on hospital day 16. Ultrasonogram of the liver and biliary tree was normal. On hospital day 18, dasatinib dose was increased to its normal dose of 100 mg daily. Liver function tests done on hospital day 22 showed that total bilirubin trended down to 1.0 mg/dL, AST trended down to 19 mg/dL, and ALT trended down to 24 mg/dL. Then, on hospital day 29, the patient developed new-onset tea-colored urine, fever, chills, bilateral leg pain, and muscle cramps. Blood cultures and urine cultures were negative. Repeat liver function tests showed the following results: total bilirubin was 2.3 mg/dL, AST was 1254 mg/dL, and ALT was 290 mg/dL. Serum creatine kinase was >100,000 U/L (reference range: 26–308 U/L).  Figure 1 shows the trend of CK and AST. Oral dasatinib and the patient's HIV medications emtricitabine-tenofovir 200 mg–300 mg once daily with dolutegravir 50 mg daily were discontinued.

## 3. Investigations

Laboratory parameters on hospital day 29 showed pancytopenia with the hemoglobin count of 7.1 g/dL, WBC count of 0.46 K/mm [3], and platelet count of 23 K/mm [3]. Chemistry showed serum creatinine of 0.9 mg/dL, total bilirubin of 2.3 mg/dL, AST of 1254 mg/dL, and ALT of 290 mg/dL. Serum creatine kinase was >100,000 U/L (reference range: 26–308 U/L). A detailed workup for the source of sepsis was negative, including blood and urine cultures. Ultrasonogram of the liver and biliary tree was repeated, and they were normal. Urinalysis was positive for large blood but negative for red blood cells (RBCs). Urine myoglobin was elevated at 220 ng/ml (reference range: 0–13 ng/ml). Hepatitis B surface antigen, hepatitis B DNA, hepatitis C antibody, and urine histoplasma antigen were all negative. Nasopharyngeal swab for influenza A and B PCR was negative.

## 4. Differential Diagnosis

Initially, elevated liver aminotransferases were suspected to be hepatitis from drugs or infection. Other possible differential diagnoses were tumor lysis syndrome and disseminated fungal infections in an immunocompromised host, influenza, or other nonspecific viral illness. When the patient started to have tea-colored urine and musculoskeletal symptoms, rhabdomyolysis came into picture.

## 5. Treatment

The patient was vigorously hydrated with intravenous fluids. Urine myoglobin was elevated at 220 ng/ml (reference range: 0–13 ng/ml). The patient's serum creatinine stayed in a range of 0.9–1.0 mg/dL during this event. As shown in Figure 1, serum creatine kinase started trending down 3 days after initiation of intravenous fluids. The patient's muscle pain, cramp, and fever resolved. On hospital day 41, his serum creatine kinase has trended down to 2026 U/L. Based on the clinical presentation, lack of other causes of muscle damage, and the temporal association, we believed that the causative agent for his rhabdomyolysis was dasatinib. The trend of creatine kinase and AST in relation to dasatinib dosing is as shown in Figure 1. The patient was started back on his HIV regimen emtricitabine-tenofovir 200 mg–300 mg once daily with dolutegravir 50 mg daily. He was also placed on oral nilotinib 400 mg daily for treatment for chronic myeloid leukemia.

## 6. Outcome and Follow-Up

He was followed up in the clinic after discharge, and he continued to be asymptomatic. His serum creatine kinase normalized to 141 U/L (reference range: 26–308 U/L) three weeks after discharge from the hospital. He is asymptomatic and tolerating his HIV medications and nilotinib 400 mg daily 1 year after hospital discharge.

## 7. Discussion

Dasatinib is a highly potent second-generation tyrosine kinase inhibitor (BCR-ABL) used in the treatment of chronic myeloid leukemia (CML). It has a 325-fold higher potency in vitro against engineered cell lines expressing nonmutated BCR-ABL compared to imatinib [5]. There have been a total of 5 case reports of rhabdomyolysis associated with use of tyrosine kinase inhibitors. Of these five, two are due to imatinib mesylate, two are due to erlotinib, and one due to dasatinib [3, 6–9]. There is another report of erlotinib-induced rhabdomyolysis due to drug interaction with simvastatin in a patient on simvastatin, both drugs being CYP 3A4 substrates [10]. In the 24-month follow-up of a phase 3 randomized trial DASISION (dasatinib versus imatinib in newly diagnosed CML), there was one patient who discontinued dasatinib treatment due to elevated skeletal creatine phosphokinase [10]. Per package insert from the manufacturer, most common adverse reactions include myelosuppression, diarrhea, headache, skin rash, fatigue, nausea, and musculoskeletal pain, but there were no reports of grade 3 or 4 adverse reaction, including rhabdomyolysis [4].

The mechanism of muscle injury from tyrosine kinase inhibitors is poorly understood. It has been suggested that TKI inhibition of platelet-derived growth factor (PDGF) and the resultant electrolyte abnormalities could lead to muscle damage [11, 12]. In our case, we are not certain if the muscle damage is from an elevated serum level of dasatinib due to the CYP 3A4-inhibiting properties of oral voriconazole. We followed the manufacturer's guidelines of the decreasing dose of dasatinib to 20 mg daily when another potent CYP 3A4 inhibitor is administered with it [4]. Another interesting aspect of this case is the elevation of aminotransferases, especially AST. This elevation is due to its release from muscle damage. Elevation of aminotransferases in patients receiving dasatinib should alert the clinicians to check for muscle damage.

Although a definite association between rhabdomyolysis and dasatinib could not be made, we considered it a case of dasatinib-induced rhabdomyolysis due to the temporal association, lack of other possible causes of severe rhabdomyolysis, resolution of the patient's symptoms, and the return of serum CK levels to normal range after withdrawal of dasatinib. The patient was in the hospital under close monitoring and did not consume any herbal medications or illicit drugs. Based on this case, we recommend being watchful for rhabdomyolysis as a complication resulting from use of dasatinib.

## Additional Points

*Learning Points.* (i) Dasatinib is a rare cause of life-threatening rhabdomyolysis. (ii) Understanding the complex interactions between dasatinib and voriconazole. (iii) Understanding the importance of checking serum creatine kinase in an asymptomatic patient with tea-colored urine and elevated serum aminotransferase levels.
